# The role of collectivism, liberty, COVID fatigue, and fatalism in public support for the zero-COVID policy and relaxing restrictions in China

**DOI:** 10.1186/s12889-024-18331-1

**Published:** 2024-03-21

**Authors:** Xiao Wang

**Affiliations:** School of Communication, Rochester Institute of Technology, 92 Lomb Memorial Drive, Rochester, NY USA

**Keywords:** COVID-19, Policy support, China, COVID fatigue, Fatalism, Cultural values

## Abstract

**Background:**

China was the last country in the world to relax COVID-19 restrictions. A successful public health policy requires public support. This analysis examined the factors associated with Chinese support for zero-COVID and relaxing COVID-19 restrictions in China.

**Method:**

Two online surveys were conducted among Chinese participants in mainland China on June 10–13 (*N* = 460) and December 2, 2022 (*N* = 450). These two samples were similar based on the participants’ demographics.

**Results:**

The results revealed that the perceived health consequences of a COVID-19 policy, perceived norms of approving a COVID-19 policy, and hope positively predicted the participants’ support for the COVID-19 policy. The results further showed that collectivism and fatalism positively predicted support for zero-COVID and negatively predicted support for relaxing restrictions. COVID fatigue was negatively associated with support for zero-COVID and positively associated with support for relaxing restrictions. Liberty positively predicted support for relaxing restrictions in June and negatively predicted zero-COVID in December 2023. It did not positively or negatively predict support for the policy adopted by the government.

**Conclusion:**

Collectivism, liberty, COVID fatigue, and fatalistic beliefs are important considerations connected to public support for a COVID-19 policy. The role of liberty was more nuanced and depended on the survey’s time and whether the government adopted the policy.

**Supplementary Information:**

The online version contains supplementary material available at 10.1186/s12889-024-18331-1.

Shortly after the initial reports of the SARS-CoV-2 virus in Wuhan, China adopted a zero-COVID policy by instituting localized lockdowns, mass testing, and contact tracing [1, 2]. For the most part in 2020 and 2021, the lockdowns were short; daily infection statistics were low, ranging from 10s to 100 [3]. That is, the effect of the zero-COVID policy was immediate and noticeable. However, the Omicron variant is more infectious but less virulent [4]. From early April to early June 2022, Shanghai experienced a city-wide lockdown to contain COVID-19 infections [5]. In November, there was a surge of infections, with 25,000 to 30,000 daily cases, despite repeated lockdowns and frequent testing in many parts of the country [3]. In late November 2022, the Western media reported protests against the strict COVID-19 policy in several major cities in China [6, 7]. Shortly afterward, China dropped its zero-COVID policy [8, 9].

The situation in China raised a question about why Chinese residents were willing to endure COVID-19 measures in 2022, which were far more stringent than those in many Western countries where compliance was lower than in China. One stream of research has shown that collectivism, emblematic of East Asian cultures, is positively associated with a willingness to follow preventive measures [10, 11]. On the other hand, libertarian values are associated with protests against COVID-19 restrictions [12] and lower compliance [13]. However, limited research has been conducted to examine Chinese support for the zero-COVID policy in 2022 or how collectivist and libertarian values were associated with their support.

Furthermore, it is reasonable to assume that Chinese residents would experience mental fatigue due to frequent lockdowns and mass nucleic acid testing. They might also hold fatalistic beliefs about COVID-19 because it is highly infectious and all other countries dropped their COVID-19 policies. Calls for abandoning zero-COVID were often based on mental fatigue and fatalism. However, existing research on COVID fatigue in China focused on healthcare workers [14, 15], and research on the role of fatalistic beliefs among the Chinese public in 2022 was limited [16].

As such, the present research examines the role of stable cultural beliefs held by individuals and other beliefs developed during the COVID-19 pandemic in their support for zero-COVID and its alternative of living with COVID-19. Notably, the present research examines public support for pandemic measures in China, which is politically and culturally different from Western countries and Asian neighbors. More accurately referred to as a semi-authoritarian regime, China strives to be responsive to public needs in exchange for political stability [17, 18]. Understanding public concern and underlying reasons can facilitate support for future disease prevention in China and offer the academic community a point to compare with the findings from other countries.

The structure of this article is as follows: First, it discusses the overall theoretical framework based on Fishbein and Ajzen’s reasoned action, whereby variables related to policy support and behavioral action are incorporated (e.g., beliefs toward health consequences, norms, and hope). Second, it discusses the role of antecedent variables such as collectivism, liberty, COVID fatigue, and fatalism. Third, the method section presents the survey timeline and procedure, demographic information of the participants, and the measures. Fourth, the results section presents moderated hierarchical analysis used to examine the role of the antecedent variables in public perceptions and support. Finally, the article discusses the results within the Chinese cultural and political contexts and offers theoretical and practical implications.

## Predicting policy support: health consequences, norms, and hope

**Fig. 1:**
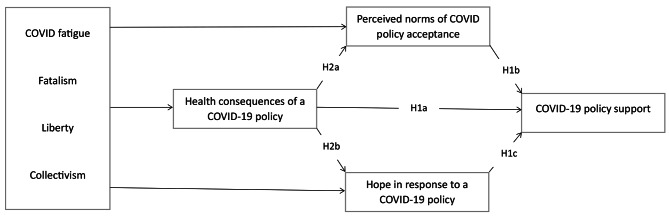
Proposed theoretical relationships *Note*. H3 and H4 predicted the total relationships between the antecedent variables (e.g., COVID fatigue) and COVID-19 policy support, which cannot be visually shown here. Health consequences, norms, and hope mediate these relationships

A working model (Fig. 1) guides the present research. Based on Fishbein and Ajzen’s theorizing [19], this model is conceptualized and complemented with hope. In its nutshell, this model states that (a) attitudes (or attitudinal beliefs), norms, and hope predict policy support and that (b) antecedent variables such as values (e.g., collectivism and liberty) and emotions (e.g., COVID fatigue) predict attitudes, norms, and hope, which then predict policy support.

Attitudes refer to individuals’ positive and negative evaluations of a behavior (or a COVID-19 policy) [19]. They are based on beliefs about a behavior, for example, the health consequences of a COVID-19 policy. Perceived norms refer to the expectations and opinions of significant others (e.g., their support for a COVID-19 policy) [19]. Norms offer meaning and guidance for human behavior. If many others perform a behavior (or approve a policy), it is assumed that such a behavior is sensible to follow (or a good policy to adopt) [20], that is, favorable attitudes toward a behavior or enacting a behavior.

This research further includes hope as an additional predictor of support for a COVID-19 policy. Hope is a positive, uplifting emotion that individuals experience when they speculate that the future can be better than the present. Hope arises when individuals expect to obtain rewards or avoid punishment in the future in an uncertain situation [21]. In particular, after having experienced frequent lockdowns and testing, Chinese residents might hope for a better situation. Because hope can be a strong motivator of people’s behaviors [21] and policy support [22], hope is postulated to predict their support for a COVID-19 policy.

We also expect that the perceived health consequences of a COVID-19 policy predict norms and hope. First, theorizing on norms states that norms guide social order and are based on what is appropriate or effective [20, 23]. That is, more appropriate, better consequences of a preventive behavior elicit stronger perceived norms. Second, individuals experience hope, a positive emotion, when they expect to obtain rewards and avoid punishment [21]. A better perceived consequence will result in greater hope. At the time of the surveys, zero-COVID and relaxing restrictions had positive and negative health consequences. Relaxing restrictions would free individuals from lockdowns and constant COVID-19 testing. However, it could result in a surge of COVID-19 infections and overwhelm hospitals, leading to a high mortality rate. It is safe to assume that individuals carried various expectations of the two COVID-19 policies and, hence, different levels of hope. Based on the preceding, the following research questions are asked.

### H1

(a) Perceived health consequences, (b) perceived norms, and (d) hope would predict support for a COVID-19 policy.

### H2

Individuals who perceived more positive health consequences of the COVID-19 approaches would (a) perceive stronger norms of supporting a COVID-19 policy and (b) feel stronger hope.

## Antecedent variables

This research includes four antecedents related to COVID-19 research: collectivism, liberty, COVID fatigue, and fatalism.

## Collectivism

Cultural values shape people’s worldviews and behavior [24]. One crucial cultural value orientation is collectivism and individualism. Those who are more collectivist (vs. individualistic) value the collective well-being of their group and put the group before the individual [25]. As such, they are willing to make sacrifices and cooperate with others to achieve collective well-being and support policies advocating collective benefits. Hofstede found that East Asian countries, including China, emphasize collectivism over individualism [26]. However, recent theorizing emphasizes the need to consider intra-country differences [27]; within a country, some individuals are more collectivist than others.

Related to COVID-19 research, several studies have shown that collectivism was positively associated with COVID-19 preventive behaviors, for example, mask-wearing and support for social distancing and lockdowns [28, 29]. Lu et al. performed a secondary analysis and examined the collectivism-individualism index and facemask-wearing percentages in the United States [28]. Lu et al. found a positive correlation between the two. Chung et al. found that cultural values were significantly related to older South Korean adults’ mask-wearing [10]. Nakayachi et al. reported that Japanese participants’ self-reported conformity to societal norms was positively associated with mask-wearing despite the limited efficacy of masks in preventing COVID-19 infections [11]. Based on the data from 98 countries, Webster et al. found that country-level collectivism was negatively associated with COVID-19 death rates [30], which is consistent with the research discussed above. However, Webster et al. found that in the United States, state-level collectivism was positively associated with COVID-19 infection and death rates and that race was a more important factor in predicting COVID-19 infection and death rates. Such results indicate that variables other than cultural values and prevention measures can influence COVID-19 infection and death rates.

Based on the research above, the present research postulates that collectivist values held by Chinese participants predicted their support for COVID-19 policies. First, because collectivists value their group’s well-being, they are more likely to value behaviors or policies that promote group benefits (i.e., greater health benefits of zero-COVID) or prevent risks to their group [31, 32]. Second, collectivists are also more sensitive to others’ opinions [33] and tend to conflate self and others [34]. Furthermore, because COVID-19 is a respiratory disease that spreads from person to person, it threatens many living in the same community. As such, collectivists should expect the community to address the issue (e.g., perceived norms) and are more likely to perceive higher norms than individualists. Leong et al. found that perceived norms positively mediated the relationship between collectivism and COVID-19 preventive behaviors [35]. Third, hope is based on the expectation that individuals can achieve their goals [36]. Individuals have agency (i.e., determination, motivation, and capability) to achieve a positive outcome. In a collectivist country such as China, agency refers to social agency reflecting the interdependence of individuals and their external environments (e.g., significant others and different situations) [34]. Collectivists are more likely than individualists to trust their group and collective ability (i.e., social agency) to achieve an outcome benefiting the group [37], leading to stronger support for preventive measures or policies.

## Liberty

The present research also examines the role of liberty in Chinese attitudes and thoughts toward COVID-19 policies. Liberty refers to “freedom from interference” or “the state of being free within society from oppressive restrictions imposed by authority on one’s way of life, behavior, or political views.” “Freedom from interference” follows Locke’s notion and focuses on individual autonomy and what individuals can do [38]. This notion is consistent with the refusal to support or recognize government interventions. Individuals with high libertarian values do not want others to interfere with them and, at the same time, do not interfere with others’ lives. Such libertarian values go against a strict zero-COVID policy and are consistent with living with COVID-19, where government interventions are minimal. Much Western commentary about zero-COVID focused on Chinese residents’ rights to liberty and autonomy.

The cultural theory of risk explains the relationship between liberty and policy support. Because libertarians want to be free from government interference, they dismiss the potential health risks a disease imposes and the associated health benefits of prevention or mitigation behaviors [39]. In other words, why is it necessary to allow government inference in the (perceived) absence of health risks and benefits? Furthermore, libertarians focus on individual rights and are not sensitive to norms postulated to predict policy support. Siegrist and Bearth reported that participants’ perceptions of liberty (i.e., opposite of communitarianism), operationalized as free from government interference, were negatively associated with their perceptions of COVID-19 risks and support for containment measures in Switzerland [40]. Limited research conducted in the United States found that individuals with higher libertarian values were less supportive of government interventions and instead advocated individual freedom [12, 41]. Taken together, liberty should negatively predict policies that restrict individual freedom and positively predict policies that allow it.

## COVID fatigue

COVID or pandemic fatigue is “the potential exhaustion, tiredness, and fatigue induced by the pandemic” (p. 2) [42]. According to the November 2, 2020 document, the World Health Organization (WHO) states that pandemic fatigue is “a reaction to sustained and unresolved adversity which may lead to complacency, alienation and hopelessness, emerging gradually over time and affected by a number of emotions, experiences and perceptions” (p. 7) [43]. The WHO states that pandemic fatigue evolves naturally and gradually within a “cultural, social, structural and legislative environment.” JØrgensen et al. surveyed participants in seven European countries (e.g., Denmark, the United Kingdom, and France) and the United States in 2020 and 2021 [42]. They found that COVID fatigue increased with time and policy stringency and when the severity of the pandemic decreased. Repeated lockdowns, other forms of restricted movements, and mass nucleic acid testing in China in 2022 could have contributed to increased COVID fatigue among Chinese residents.

COVID fatigue played a role in pandemic prevention and policy support. Individuals experiencing COVID fatigue are mentally and physically tired of paying attention to COVID-19 messages and performing preventive behaviors [44]. Over time, individuals became desensitized by COVID-19 news and statistics and tired of performing preventive behaviors. The initial eagerness and determination to perform preventive measures decreased, especially when the costs were much higher than the returns [42]. Overall exhaustion increased when it became more difficult to contain a virus and when the end of the pandemic was not known. As a result, COVID fatigue was associated with irritation and demotivation to perform COVID-19-related preventive behaviors [43, 44]. Furthermore, because central or local governments often mandated pandemic prevention, COVID fatigue also contributed to anger toward the government and could fuel political discontent [42].

For example, Morgul et al. conducted a cross-sectional survey in Turkey [45]. They reported that individuals who were not fatigued showed more positive attitudes toward and greater satisfaction with mandated preventive measures and were more optimistic that COVID-19 could be controlled than the fatigued participants. JØrgensen et al. found that pandemic fatigue was positively associated with concerns about democratic rights, opposition to COVID-19 restrictions, and support for protests [42]. Kim et al. conducted a population-based longitudinal survey with participants in Hong Kong [46]. They found that participants showed a decline in compliance with pandemic prevention measures (e.g., avoiding social gatherings or international travel). In sum, COVID fatigue is characterized by exhaustion and tiredness. Fatigued individuals would be desensitized toward COVID-19 statistics, hold less favorable attitudes, and be less optimistic about preventive measures, which, in turn, would be negatively associated with policy support.

## Fatalism

Drawing on the previous theorizing, this paper defines fatalism as the belief that contracting a disease and dying are difficult to avoid [47, 48]. In general, fatalistic beliefs are characterized by (a) a lack of personal control of a disease and (b) perceptions of luck or fate in avoiding or contracting a disease. These beliefs are subsequently associated with hopelessness and stress, demotivation to change an unhealthy behavior, and, consequently, adverse behavioral outcomes in many cancer-related behaviors and outcomes [49].

Previous research on fatalistic beliefs about COVID-19 and preventive behaviors was conducted early in the COVID-19 pandemic. For example, Akesson et al. reported that participants who believed COVID-19 was more infectious were less willing to take preventive measures [50]. Hayes and Clerk (2021) conducted an experiment with participants in the United States. Hayes and Clerk found that fatalistic beliefs were negatively associated with intentions to support mitigation after viewing a message [51]. Jimenez et al. revealed a similar relationship between fatalistic beliefs about COVID-19 (e.g., COVID-19 was associated with death) and preventive behaviors. In addition to the relationship reported above, Akesson et al. (2022) reported that fatalistic beliefs were negatively associated with hope and optimism [50]. In general, individuals with fatalistic beliefs believe that they do not have control over a disease; as such, performing a preventive behavior is not beneficial.

The Omicron variant was much more infectious, although less virulent, and caused waves of infections in many countries. Even with lockdowns, mass testing, and quarantine, China experienced 16,000 cases on June 15, 2022, and more than 35,000 cases daily in November 2022 [3]. As such, the virus appeared to be much less controllable, and the costs of lockdowns and mass testing were more significant than before. That is, it was natural for some Chinese residents to develop beliefs in favor of abandoning the fight. As such, individuals who had stronger fatalistic beliefs would be more likely to support policies to relax restrictions instead of the zero-COVID policy.

As such, this research proposes the following hypotheses:

### H3

(a) Collectivism was positively associated with support for zero-COVID, and (b) liberty, (c)

COVID fatigue, and (d) fatalism were negatively associated with support for zero-COVID.

### H4

(a) Collectivism was negatively associated with support for relaxing restrictions, and

(b) liberty, (c) COVID fatigue, and (d) fatalism were positively associated with support for relaxing restrictions.

Because this research surveyed participants in June and early December 2022, it presents changes in public attitudes, norms, and hope between June and early December 2022. It also explores the possible interaction effects between the predictor variables and the time of the surveys on policy support.

## Method

This analysis used part of the data collected from two surveys conducted in mainland China between June 10 and 13, 2022, shortly after Shanghai endured a two-month lockdown, and on December 2, 2023, after China relaxed its COVID-19 restrictions in late November 2022. Rochester Institute of Technology’s Institutional Review Board approved the survey protocol in May 2022.

The data were collected via Credamo’s survey platform. Credamo is a survey website with three million panel members in all the provinces and directly administered cities in mainland China. According to its user manual [52], the panel members were recruited from offline channels: customers/shoppers, company employees, college students and staff, and those who previously completed its offline surveys. For both surveys, the response rate was approximately 33%. The final samples consisted of 460 and 450 respondents.

Regarding the participants’ demographics, 49.7% were females, and 50.3% were males. Most participants were of Han ethnicity (98.0%), and the remaining were of other ethnicities. The means and standard deviations, age, year of education, and annual income were 31.1 (*SD* = 8.40), 15.7 (*SD* = 2.4), and RMB115,400 or USD15,877 (*SD* = RMB78,170 or USD10,659), respectively. Participants came from Guangdong (14.1%), Shandong (13.2%), Jiangsu (6.4%), Jiangxi (4.0%), Hebei (4.7%), Zhejiang. (5.1%), and others. Qinghai and Xizang were the only two provinces in mainland China not represented. Participants’ occupations varied: Most participants were employed. The unemployed group represented 0.4% of the participants. Students consisted of 15.4% of the sample. Detailed demographic information of the two samples was presented in the online supplementary Table S1.

### Measures

Tables 1 and 2 show the items for the measures used in the analysis. All the responses ranged from 1 (*strongly disagree*) to 7 (*strongly agree*). Note that another paper based on this dataset examined the role of risk perceptions and media use [53]: Perceived health consequences were the only overlapping variable in the two papers other than the demographic variables.

**Table 1:** Confirmatory factor analysis of items used to measure the antecedent variables

Variable and question	Standardized factor loading
COVID fatigue (AVE = 0.61, CR = 0.89)	
I’m tired of following COVID lockdowns and quarantine	0.76
I’m strained from following all the COVID recommendations	0.71
I’m losing my spirit in the fight against COVID-19	0.69
I’m tired of COVID-19 discussions	0.88
I’m sick of hearing about COVID-19	0.85
Fatalism about getting COVID-19 (AVE = 0.60, CR = 0.82)	
Other countries’ “lying flat” COVID policies make it difficult for us to achieve zero-COVID	0.85
Other countries’ COVID policies make me feel helpless	0.79
The Omicron variant is too infectious and difficult to control	0.67
Liberty (AVE = 0.64, CR = 0.84)	
People should be free to decide what they want to follow	0.80
People should be free to enjoy their life as they see fit	0.91
The government should not interfere too much in our everyday lives	0.67
Collectivism (AVE = 0.45, CR = 0.71)	
Everyone should contribute to their group	0.65
I feel good when I cooperate with others	0.66
Everyone should make some sacrifices for a better world	0.69

Note. *N* = 910. AVE = average variance extracted. CR = composite reliability. Model fit statistics: χ^2^ = 421.3, *df* = 71, comparative fit index = 0.92, root mean square error of approximation (RMSEA) = 0.074, 90% CI of RMSEA (0.067 0.080), root mean square residual = 0.08

**Table 2:** Items used to measure the variables related to zero-covid and lying flat: confirmatory factor analysis results

Variable and question	Standardized factor loading*	
Positive health consequences (AVE = 0.56, CR = 0.88) (AVE = 0.77, CR = 0.95)^a^	Zero-COVID	Lying flat
In general, the zero-COVID policy/relaxing COVID restrictions can….		
prevent people from being infected	0.76	0.90
protect my health	0.82	0.93
protect others’ health	0.79	0.93
protect my family’s health	0.78	0.93
prevent COVID-19 outbreaks	0.65	0.87
help avoid crowding hospitals	0.67	0.68
Subjective norms (AVE = 0.67, CR = 0.86) (AVE = 0.84, CR = 0.94)		
My family supports it	0.89	0.93
My neighbor/community supports it	0.74	0.90
The majority of the Chinese support it	0.81	0.92
Hope (in response to a COVID policy) (AVE = 0.69, CR = 0.90) (AVE = 0.85, CR = 0.96)		
I feel optimistic	0.82	0.90
I think of the positive side	0.84	0.92
I believe my life will be better	0.81	0.93
I feel hopeful	0.86	0.93
Support for a COVID policy (AVE = 0.73, CR = 0.92) (AVE = 0.88, CR = 0.97)		
it is acceptable	0.84	0.92
we should support it	0.87	0.95
it should be adopted	0.85	0.94
it’s a good choice	0.85	0.94

*Note: N* = 910. AVE = average variance extracted. CR = composite reliability. ^a^ AVE and CR for zero-COVID (left) and relaxing restrictions (right). Model fit statistics: χ^2^ = 893.4, *df* = 1246, comparative fit index = 0.98, root mean square error of approximation (RMSEA) = 0.029, 90% CI of RMSEA: [0.026 0.033], and root mean squared residual = 0.027. Prompts about either zero-COVID or relaxing restrictions preceded all the above questions

Tables 1 and 2 show that the average variance extracted (AVE) and composite reliabilities were greater than 0.50 and 0.70, respectively, showing satisfactory convergent validity of the items. Note that collectivism had an AVE of 0.45, lower than 0.50. However, Fornell and Larcker state that AVE is a conservative measure of convergent validity and that the researcher may conclude that the convergent validity of a scale is adequate based on composite reliability (p. 46) [54]. The composite reliability of collectivism is 0.71. The AVE of each scale was greater than the squared correlations between that scale/variable and other variables, showing satisfactory discriminant validity.

Collectivism was measured by three items adapted from the literature [25, 55], for example, “everyone should contribute to their group.” The Cronbach alpha was 0.72.

Liberty was measured by three items adapted from Iyer et al. [56] (2012). For example, “people should be free to decide what they want to follow.” The Cronbach alpha was 0.83.

COVID fatigue was measured by five items adapted from Rodriguez-Blazquez et al. [57]. The questions include, for example, “I’m tired of COVID discussions.” The Cronbach alpha was 0.89.

Fatalistic beliefs about contracting COVID-19 were measured by three items adapted from Esparza et al.’s general fatalism scale and edited to reflect the specific COVID-19 situation [58]. The items included “The Omicron variant is too infectious and difficult to control” and “Other countries’ COVID-19 policies make me feel helpless.” The Cronbach alpha was 0.81.

Perceived health consequences of zero-COVID and relaxing restrictions were each measured by six questions reflecting COVID-19 concerns among the public and in the media. For example, “All in all, the zero-COVID policy can prevent people from getting infected.” The same six questions were used to measure the perceived health consequences of relaxing COVID-19 restrictions by replacing “zero-COVID” with “relaxing COVID restrictions.” For example, “All in all, relaxing COVID restrictions can prevent people from getting infected.” The alpha coefficients for the measures were 0.88 (zero-COVID) and 0.95 (relaxing restrictions).

Perceived norms of supporting zero-COVID were measured by three items adapted from Fishbein and Ajzen [19]. For example, “Regarding the zero-COVID policy, my family supports it.” Perceived norms of supporting relaxing restrictions were measured by the same three items by replacing zero-COVID with “relaxing restrictions.” The alpha coefficients were 0.86 and 0.94.

Hope about zero-COVID was measured by four items selected from the hope literature [59]. The items included “Thinking about the consequences of the zero-COVID policy, I feel hopeful.” The same four items were used to measure hope toward relaxing restrictions by replacing “the zero-COVID policy” with “relaxing COVID restrictions.” The alpha coefficients were 0.90 and 0.90, respectively.

Support for zero-COVID was measured by four items. The set of questions began with “regarding zero-COVID…,” followed by “we should support it” and three additional items (see Table 2). The same items measured support for relaxing COVID-19 restrictions by replacing “zero-COVID” with “relaxing COVID restrictions.” The alpha coefficients were 0.91 and 0.97.

Questions related to socio-demographic variables were placed at the end of the questionnaire.

## Results

### Descriptive statistics

Table 3 presents the descriptive statistics and changes in Chinese attitudes, norms, hope, and support for a COVID-19 policy between June and early December 2022. By early December, Chinese participants held more favorable attitudes, norms, and hope about zero-COVID than relaxing restrictions. However, attitudes, norms, and hope became less favorable toward zero-COVID and more favorable toward relaxing restrictions between June and December 2022.

**Table 3:** Independent-samples t tests comparing participants’ values and attitudes toward covid policies

	June 10–13 survey (*N* = 460)	Dec. 2 survey (*N* = 450)	t value	df	p value	Lower of 95% CI	Upper of 95% CI of
Fatigue	2.84	3.15	-3.46	883.6	0.001	-0.49	-0.13
	1.25	1.45					
Fatalism	4.50	4.32	1.92	904.2	0.055	0.00	0.38
	1.43	1.49					
Liberty	3.59	3.87	-3.12	908.0	0.002	-0.46	-0.11
	1.37	1.37					
Collectivism	5.87	5.85	0.34	908.0	0.736	-0.12	0.09
	0.82	0.78					
Health consequences of zero-COVID	6.07	5.85	3.75	835.2	0.000	0.10	0.33
	0.74	0.98					
Health consequences of relaxing restrictions	2.26	3.11	-8.92	866.3	0.000	-1.04	-0.67
	1.29	1.58					
Perceived norms for supporting zero-COVID	6.14	5.59	7.60	835.6	0.000	0.40	0.69
	0.92	1.22					
Perceived norms for relaxing restrictions	2.32	3.76	-13.04	867.8	0.000	-1.65	-1.22
	1.49	1.81					
Hope in response to zero-COVID)	5.90	5.51	5.34	834.7	0.000	0.25	0.53
	0.93	1.24					
Hope in response to relaxing restrictions	2.69	4.00	-11.82	894.1	0.000	-1.54	-1.10
	1.59	1.76					
Support for zero-COVID	5.96	5.47	6.33	846.9	0.000	0.34	0.64
	1.02	1.31					
Support for relaxing restrictions	2.39	3.74	-11.60	869.7	0.000	-1.57	-1.12
	1.58	1.91					

### Direct predictors of policy support

This research adopted moderated multiple regression to examine direct and total relationships (Fig. 1). The overall model was similar to PROCESS MACRO model 81 but incorporated survey time as a moderator. The initial analysis showed that survey time did not interact with COVID fatigue or fatalism; these two interaction terms were then removed from the final analysis. Table 4 provides the direct and total relationships between the predictor variables and public policy support. Demographic variables and political philosophy were controlled for.

**Table 4:** Direct and total relationships between the predictor variables and support for covid policies

	Support for Zero-COVID																Support for relaxing restrictions														
	Direct Effect								Total effect								Direct effect								Total effect						
Predictor	*B*		*SE*		*b*				*B*		*SE*		*b*				*B*		*SE*		*b*				*B*		*SE*		*b*		
Control variable																															
Gender (1 = male, 2 = female)	-0.04	0.04		− 0.02				-0.11		0.06		− 0.05+				0.04		0.06		0.01				0.09		0.11		0.02			
Age (year)	0.00	0.00		− 0.03				0.00		0.00		0.01				0.01		0.00		0.03				0.02		0.01		0.07^*^			
Education (year)	-0.01	0.01		− 0.01				-0.01		0.01		− 0.02				0.01		0.01		0.01				-0.02		0.02		− 0.03			
Annual income (1 = 10 K)	0.01	0.00		0.03				0.00		0.00		− 0.01				0.00		0.00		0.01				0.01		0.01		0.03			
Political philosophy	-0.07	0.02		− 0.08^***^				-0.08		0.02		− 0.10^***^				0.02		0.02		0.01				0.18		0.04		0.14^***^			
Time of survey (0 = June, 1 = December)	-0.07	0.05		− 0.03				-0.35		0.06		− 0.15^***^				0.04		0.06		0.01				1.18		0.11		0.32^***^			
Antecedent variable																															
COVID fatigue	-0.09	0.02		− 0.10^***^				-0.35		0.03		− 0.40^***^				0.10		0.03		0.07^**^				0.41		0.05		0.30^***^			
Fatalism	0.01	0.02		0.02				0.08		0.02		0.10^***^				-0.01		0.02		− 0.01				-0.15		0.04		− 0.12^***^			
Liberty	-0.02	0.02		− 0.02				-0.05		0.03		− 0.06				0.05		0.03		0.04				0.21		0.06		0.15^***^			
Collectivism	0.12	0.04		0.08^**^				0.48		0.06		0.31^***^				-0.13		0.05		− 0.05^***^				-0.36		0.10		− 0.15^***^			
Interaction effect																															
Liberty × time	-0.09	0.03		− 0.07^**^				-0.08		0.05		− 0.07^*^				-0.03		0.04		− 0.01				-0.18		0.08		− 0.09^*^			
Collectivism × time	-0.13	0.06		− 0.06^**^				-0.05		0.08		− 0.02				-0.06		0.07		− 0.02				-0.015		0.14		− 0.00			
Mediating variable																															
Health consequences of COVID policy	0.17	0.03		0.13^***^				0.54		0.04		0.40^***^				0.19		0.03		0.15^***^				0.76		0.03		0.61^***^			
Perceived norm of COVID policy support	0.41	0.03		0.38^***^												0.38		0.03		0.37^***^											
Hope	0.34	0.03		0.31^***^												0.41		0.03		0.39^***^											

Note. *N* = 910 (*N* = 460 in June, *N* = 450 in December 2022). Model statistics for zero-COVID (direct effects): block 1 *R*^2^ = 0.079, *F*(6, 903) = 12.96, *p* < .001; block 2 *R*^2^ = 0.345, *F*(4, 899) = 134.95, *p* < .001; block 3 *R*^2^ = 0.002, *F*(2, 897) = 1.74, *p* =.176; block 4 *R*^2^ = 0.288, *F*(3, 894) = 300.49, *p* < .001. Model statistics for relaxing restrictions (direct effects): block 1 *R*^2^ = 0.162, *F*(6, 903) = 29.11, *p* < .001; block 2 *R*^2^ = 0.145, *F*(4, 899) = 46.88, *p* < .001; block 3 *R*^2^ = 0.004, *F*(2, 897) = 2.75, *p* =.176; block 4 *R*^2^ = 0.491, *F*(3, 894) = 740.9, *p* < .001

For H1a-c, the participants’ perceived positive health consequences of the zero-COVID policy (β = 0.13, *p* < .001), perceived norms about zero-COVID (β = 0.38, *p* < .001), and associated hope (β = 0.31, *p* < .001) predicted their support for zero-COVID.

For H1a-c, regarding support for relaxing restrictions, the participants’ perceptions of positive health consequences of relaxing COVID-19 restrictions (β = 0.15, *p* < .001), perceived norms about relaxing restrictions (β = 0.37, *p* < .001), and associated hope (β = 0.39, *p* < .001) predicted their policy support.

For H2, perceived health consequences of zero-COVID positively predicted (a) perceived norms (β = 0.40, *p* < .001) and (b) hope induced by zero-COVID (β = 0.37, *p* < .001). The total effects of perceived health consequences on support for zero-COVID were 0.40 (*p* < .001).

Furthermore, perceived health consequences of relaxing restrictions positively predicted perceived norms (β = 0.62, *p* < .001) and hope induced by relaxing restrictions (β = 0.56, *p* < .001). The total effect of perceived health consequences on support for zero-COVID was 0.61 (*p* < .001).

### Total relationships between antecedent variables and policy support

For H3a, collectivism was positively associated with support for zero-COVID (β = 0.31, *p* < .001).

For H3b, however, an interaction effect was observed regarding the predictive power of liberty: Liberty was not a significant predictor of support for zero-COVID in June 2022 when the government enacted the policy (β = − 0.05, *p* =.089). However, it negatively predicted support for zero-COVID after the government dropped the policy (β = − 0.13, *p* < .001).

For H3c, the results showed that COVID fatigue was negatively associated with support for the zero-COVID policy (β = − 0.40, *p* < .001). Contrary to the prediction (H3d), fatalism positively predicted support for zero-COVID (β = 0.10, *p* < .001), albeit with a small effect size.

For H4a, collectivism was negatively associated with support for relaxing restrictions (β = − 0.15, *p* < .001).

For H4b, an interaction effect was observed regarding the predictive power of liberty. Liberty significantly predicted support for relaxing restrictions in June 2022 when the government chose zero-COVID (β = 0.15, *p* < .001). Liberty was unrelated to support for relaxing restrictions after the government adopted this policy (β = 0.03, *ns*).

For H4c, COVID fatigue was positively associated with support for relaxing restrictions (β = 0.30, *p* < .001). Contrary to the prediction (H4d), fatalism negatively predicted support for relaxing restrictions (β = − 0.12, *p* < .001). The effect size was small.

For interested readers, Figures S1 and S2 in the online supplementary materials provide additional information about the direct paths between antecedent variables, mediating variables, and policy support.

## Discussion

The present research examined Chinese residents’ changing attitudes toward and support for COVID-19 policies in June and early December 2022 and the role of cultural values, COVID fatigue, and fatalism in predicting policy support. Table 5 presents a summary of the hypotheses and whether they were supported.

**Table 5:** Support (or lack of) for hypotheses and explanations

Hypothesis Number	Hypothesis	Supported (or not) and explanation
H1a	Perceived positive health consequences of a COVID policy are associated with stronger support for the COVID policy	Supported
H1b	Perceived norms of supporting a COVID policy is associated with stronger support for the COVID policy	Supported
H1c	Hope induced by a COVID policy is associated with higher support for the COVID policy	Supported
H2a	Perceived positive health consequences of a COVID policy are associated with perceived norms of supporting that policy	Supported
H2b	Perceived positive health consequences of a COVID policy are associated with stronger hope	Supported
H3a	Collectivism is positively associated with support for zero-COVID	Supported
H3b-d	(b) liberty, (c) COVID fatigue, and (d) fatalism are negatively associated with support for zero-COVID.	H3b: not supported in June 2022 survey, supported in December 2022 survey H3c: supported H3d: not supported: opposite, but weak relationship
H4a	Collectivism is negatively associated with support for relaxing restrictions	Supported
H4b-d	(b) liberty, (c) COVID fatigue, and (d) fatalism are positively associated with support for relaxing restrictions.	H3b: supported in the June 2022 survey, not supported in the December 2022 survey H3c: supported H3d: not supported: opposite, but weak relationship

### Theoretical discussion

First, despite the changes between June and early December 2022, the psychological reasons for supporting the two COVID-19 approaches remained unchanged (Table 5), with some exceptions. H1 found that participants’ support for a COVID-19 policy was directly predicted by their perceptions of positive health consequences, norms, and hope toward a COVID-19 policy. This analysis (H1 and H2) showed that the effects of health consequences were direct and mediated by norms and hope. These results showed that the immediate predictors of either policy support were the same. If participants perceived more positive health consequences of zero-COVID (or relaxing restrictions), they were more likely to perceive higher norms of approving this policy and higher hope, which were positively associated with their support for zero-COVID (or relaxing restrictions). The results provide evidence that the reasons for public support for a health policy can be multifaceted and that the theoretical relationships can be more complicated than the parallel relationships specified by several theoretical models of health behaviors (e.g., the health belief model) [60]. Future research should consider such complexities.

Second, the results illustrate the role of a perceived public opinion climate (i.e., perceived norms of approving a health policy) in Chinese support for a COVID-19 policy. Research on bandwagon effects states that the public adopts a behavior or belief if they believe others have adopted a similar belief or behavior [61]. The primary thesis of bandwagon effects is that individuals do so to fit in or gain access to a social group. The present research reveals that such beliefs can result from the perceived positive benefits of a policy; that is, participants can project others’ opinions based on their perceptions of health benefits. Furthermore, perceptions of others’ opinions can come from mass media and interpersonal communication [62]. My ongoing ethnographic research showed that although the Chinese public held mixed opinions, the public was generally aware of the COVID-19 infection rates and statistics in other countries and was fearful of the impact of COVID-19 on the elderly population in 2022. Perceived norms or public opinion climates could be more pronounced in China when individuals had no reliable personal experience to share.

Third, the results for H3 and H4 further prove the importance of examining the antecedent variables. Although the antecedent variables (i.e., collectivism, fatigue, and fatalism) all predicted support for zero-COVID and relaxing restrictions, these antecedent variables consistently showed opposite directions in predicting the two policies based on total effects. H3a and H4a showed that more collectivist people were more likely to support zero-COVID but less likely to support relaxing restrictions. These results are consistent with the research conducted in Hong Kong, Japan, and South Korea, which showed that more collectivist individuals were more likely to follow pandemic prevention measures [10, 11]. These results are also consistent with the cultural theory of risk whereby more collectivist individuals are more likely to scale up the risks due to their concerns about collective benefits and thus support policies to prevent potential risks to their groups. H3c and H4c showed that those experiencing more fatigue were less likely to support zero-COVID and more likely to support relaxing restrictions, which is consistent with previous research and indicates the need to address pandemic fatigue.

For H3d and H4d, fatalistic beliefs showed weak and positive relationships with policy support. The direction of the relationships was not previously predicted. Research conducted on COVID-19 elsewhere [50, 63] showed that participants holding fatalistic beliefs had lower intentions to perform COVID-19 preventive behaviors. The present results showed the opposite direction: Those with stronger fatalistic beliefs (e.g., difficulty in avoiding COVID-19) were more likely to advocate zero-COVID and less likely to support relaxing restrictions. Such results indicate that for public health issues requiring individual and group interventions, some individuals may resort to group interventions when believing they are defenseless at the individual level. However, the present research cannot ascertain whether such results resulted from the collectivist culture and public trust in government interventions experienced in 2020 and 2021. Future research should consider the type of preventive measures (e.g., individual behavior vs. government mandates) and the role of culture and possibly communitarianism philosophy (i.e., advocating government interventions).

The role of liberty in predicting public support for either policy was nuanced (H3b and H4b): Liberty was positively associated with relaxing restrictions in June but not in early December 2022. It was negatively associated with support for zero-COVID in December 2023 but not June 2022. Furthermore, liberty was not associated with opposing or supporting the government’s chosen policy in June (zero-COVID) or December 2022 (i.e., relaxing COVID-19 restrictions), respectively. First, these results indicate that being more libertarian does not mean that the libertarian participants categorically oppose the government’s policies regardless of whether the policy allows or restricts freedom to citizens. Second, the more libertarian participants did not consistently endorse a lax policy or oppose a restrictive policy. There are competing schools of thought on the consequences of zero-COVID and relaxing restrictions in June and December 2022. Such uncertainties (e.g., positive or negative health benefits of zero-COVID or relaxing restrictions) might have made the zero-COVID, although restrictive, a wise choice in June and relaxing restrictions, although allowing freedom, a risky choice in early December 2022. In these situations, individuals with high libertarian values might judge multiple factors when supporting or opposing COVID-19 policies. It was also possible that diverse opinions existed among those with a high libertarian value, resulting in no consistent patterns in predicting their COVID-19 policy support. Taken together, the present research reveals (Table 5) that the role of fatalism and liberty in public support for zero-COVID and its alternative could be different from that in other countries. Future research should further examine whether the characteristics of a disease and associated preventive measures, contextual factors, and cultural values contribute to the nuanced results.

### Further discussion of the results within the Chinese context

There has been a rise in nationalism in China in recent years. Yang observed COVID-19 nationalism in 2020, whereby Chinese residents supported Chinese government policy and opposed those who criticized the Chinese government [64]. It is possible that COVID-19 nationalism also contributed to public support for zero-COVID in 2022. However, the present dataset did not include a measure of COVID-19 nationalism. Furthermore, the present dataset does not address whether political authoritarianism and censorship influenced the public acceptance of a COVID-19 policy. However, systemic political repression generally deters protests [65] and is unlikely to lead to a higher level of support, as reported in this article. For example, political censorship online (a form of repression) would make netizens less likely to speak up or make explicit political statements. It would not make them foster favorable opinions toward the issue they protest. However, the link between political repression and public policy support can be complex, a question for future research.

Table 3 provides the means and standard deviations of the variables, including public support for the two COVID-19 policies. For example, 95% of data for public support for zero-COVID and relaxing restrictions in December 2022 were in the ranges of [2.9, 7.0] and [1.0, 7.0], respectively. The results showed that Chinese public support for zero-COVID was favorable but not monolithic. A semi-authoritarian country, China allows some free speech and tolerates criticism that does not challenge the party’s legitimacy. In addition to public health research conducted from the standpoints of cultural psychology and individual differences, future research should tackle the political and sociological aspects of pandemic responses. For example, how did Chinese residents voice their concerns about zero-COVID under China’s political system? Ethnographic research during the pandemic or using archived social media posts can provide concrete examples and detailed understanding of the interplay among politics, culture, and pandemic responses.

### Implications for public health and political support

Policies to enact or relax COVID-19 restrictions and subsequent communications should consider beliefs about health consequences, perceived norms (i.e., public opinion climate), and hope. Perceived health consequences are the root of perceived norms and hope. Thus, when considering abandoning a restrictive policy at an appropriate time, public health professionals should discuss the milder health consequences of disease and foster an opinion climate conducive to public support for reversing the zero-COVID policy. Admittedly, such policy changes and public education should be based on scientific calculations of the associated risks.

The results show the role of values, COVID fatigue, and fatalistic beliefs in predicting public support. Despite the reports of protests and public fatigue, the present research has shown minor changes in COVID fatigue and fatalistic beliefs between June and early December 2022 and stronger support for the zero-COVID policy than relaxing restrictions.

Public health officials should understand that cultural values, such as collectivism, are stable and will not change within a short period. These results, however, can help us understand why Chinese residents were more willing to endure restrictive COVID-19 policies than those in Western countries and why relaxing restrictions received less support than zero-COVID. Public health officials should aim to change other psychological correlates to change public support of a COVID-19 policy, for example, the perceived health consequences of COVID-19 discussed in the preceding and possibly the waning severity of COVID-19. Such information was widely shared in the media. In the weeks after the restrictions were dropped, the surge of COVID-19 infections and subsequent recovery provided Chinese citizens with a venue to better understand the severity of COVID-19 infections. It was later observed in January 2023 that the public largely accepted and enjoyed life without COVID-19 restrictions.

## Limitations and conclusion

First, the data were collected from samples provided by Credamo. In general, online samples are not probability-based and do not represent the population. The number of participants from China’s mega-cities or rural areas was small. However, these samples were drawn from many urban dwellers more affected by COVID-19 restrictions than rural dwellers and were generally comparable between the two waves. Second, although the relationships within each survey are correlational, the longitudinal nature of the two samples allows us to track the changes (Table 4) and indicates that the stage of the pandemic (i.e., time), policy change, and circumstances may be factors in public support. Third, between late November 2022 and January 2023, the COVID-19 situation and public attitudes toward and support for China’s new COVID-19 policies rapidly changed. It is not possible to survey the participants retrospectively. However, future research can examine China’s public response to the policy change in 2022 based on archived social media sentiments.

This analysis has shown the role of immediate predictors and antecedent variables (e.g., collectivist values, liberty, fatigue, and fatalism) in Chinese support for two COVID-19 policies. Such an analysis explains why Chinese residents are willing to support public health measures and how to respond to future disease outbreaks in China.

### Electronic supplementary material

Below is the link to the electronic supplementary material.

Supplementary Material 1

## Data Availability

The datasets used and analysed during the current study are available from the corresponding author upon reasonable request.
