# Supplementary material for: The role of collectivism, liberty, COVID fatigue, and fatalism in public support for the zero-COVID policy and relaxing restrictions in China
Source: BMC Public Health. 2024 Mar 21;24:873. doi: 10.1186/s12889-024-18331-1 (PMC10956218; doi:10.1186/s12889-024-18331-1)
Supplement: Supplementary file 1 — Supplementary Material 1 [file 12889_2024_18331_MOESM1_ESM.docx]

**Online Supplementary Materials for *BMC Public Health***

The Role of Collectivism, Liberty, COVID Fatigue, and Fatalism

in Public Support for the Zero-COVID Policy and Relaxing Restrictions in China

Xiao Wang

Rochester Institute of Technology

Table of Contents

**Contents**

[Table S1. *Participants’ Demographic Information* 2](#_Toc156677309)

[Table S2. *Means, Standard Deviations, and Pearson Correlations of the Variables* 3](#_Toc156677310)

[Figure S1. *Path Diagrams of the Variables Predicting Chinese Public Support for Zero-COVID* 4](#_Toc156677311)

[Figure S2. *Path Diagrams of the Variables Predicting Chinese Public Support for Relaxing Restrictions* 5](#_Toc156677312)

# **Table 1.** *Participants’ Demographic Information*

|  | June 2022 | December 2022 | Combined |
| --- | --- | --- | --- |
|  | *n* = 460 | *n* = 450 | *N* = 910 |
|  | Mean (SD) | Mean (SD) | Mean (SD) |
| Age | 30.83 (8.37) | 31.44 (8.43) | 31.14 (8.40) |
| Years of education | 15.70 (2.88) | 15.66 (2.89) | 15.68 (2.44) |
| Annual income (RMB) | 109,900 (73,800) | 121,000 (82,100) | 115,400 (78,170) |
| Political philosophy (1 = *conservative*, 7 = *liberal*) | 4.84 (1.30) | 4.60 (1.51) | 4.72 (1.42) |
|  | % | % | % |
| Gender |  |  |  |
| Female | 48.0% | 51.3% | 50.3% |
| Male | 52.0% | 48.7% | 49.7% |
| Ethnicity |  |  |  |
| Han ethnicity | 97.4% | 98.7% | 98.0% |
| Other ethnicities | 2.6% | 1.3% | 2.0% |
| Occupation |  |  |  |
| Administrative personnel | 19.6% | 24.4% | 22.0% |
| Professional (e.g., teacher, attorney, or doctor) | 17.0% | 18.2% | 17.6% |
| Support personnel | 16.1% | 13.6% | 14.8% |
| Student | 15.9% | 14.9% | 15.4% |
| Technology, research, & development | 15.0% | 14.2% | 14.6% |
| Marketing/customer service | 9.6% | 8.5% | 9.1% |
| Accounting | 5.2% | 4.2% | 4.7% |
| Factory/production worker | 5.0% | 4.4% | 4.7% |
| Unemployed | 0.4% | 0.4% | 0.4% |
| Top province or directly administered city |  |  |  |
| Guangdong | 15.9% | 12.2% | 14.1% |
| Shandong | 11.7% | 14.7% | 13.2% |
| Jiangsu | 6.7% | 6.0% | 6.4% |
| Jiangxi | 6.1% | 1.8% | 4.0% |
| Hebei | 5.0% | 4.4% | 4.7% |
| Zhejiang | 4.8% | 5.3% | 5.1% |
| Shanxi | 4.1% | 3.8% | 4.0% |
| Guangxi | 3.9% | 4.2% | 4.1% |
| Sichuan | 3.7% | 3.6% | 3.6% |
| Henan | 3.7% | 3.8% | 3.7% |
| Hubei | 3.7% | 3.8% | 3.7% |
| Shanghai | 2.8% | 3.8% | 3.3% |
| Beijing | 2.8% | 3.1% | 3.0% |
| Other provinces | 25.1% | 29.5% | 27.1% |

*Note*. The demographic information was also presented in Table 1 in Wang X. How the Chinese’s attitudes toward COVID-19 policies changed between June and early December 2022: risk perceptions and the uses of mainstream media and WeChat. SSM - Popul Health. 2023; 23:101467. <https://doi.org/10.1016/j.ssmph.2023.101467>. Published by Elsevier.

# **Table 2.** *Means, Standard Deviations, and Pearson Correlations of the Variables*

| Variable | 1 | 2 | 3 | 4 | 5 | 6 | 7 | 8 | 9 | 10 | 11 | 12 | 13 | 14 | 15 | 16 | 17 | 18 |
| --- | --- | --- | --- | --- | --- | --- | --- | --- | --- | --- | --- | --- | --- | --- | --- | --- | --- | --- |
| 1. Gender | - |  |  |  |  |  |  |  |  |  |  |  |  |  |  |  |  |  |
| 2. Age | -.03 | - |  |  |  |  |  |  |  |  |  |  |  |  |  |  |  |  |
| 3. Education (year) | .04 | .06 | - |  |  |  |  |  |  |  |  |  |  |  |  |  |  |  |
| 4. Annual income (1 = RMB 10,000) | .12 | .35 | .28 | - |  |  |  |  |  |  |  |  |  |  |  |  |  |  |
| 5. Political philosophy | -.03 | -.08 | .04 | .03 | - |  |  |  |  |  |  |  |  |  |  |  |  |  |
| 6. Survey time (0 = June, 1 = December) | .03 | .04 | -.01 | .07 | -.09 | - |  |  |  |  |  |  |  |  |  |  |  |  |
| 7. Collectivism | .01 | .11 | .02 | .12 | .00 | -.01 | - |  |  |  |  |  |  |  |  |  |  |  |
| 8. Liberty | -.03 | -.10 | -.02 | -.04 | .10 | .10 | -.23 | - |  |  |  |  |  |  |  |  |  |  |
| 9. COVID fatigue | .02 | -.12 | -.04 | -.21 | .07 | .11 | -.39 | .40 | - |  |  |  |  |  |  |  |  |  |
| 10. Fatalism | -.03 | -.03 | -.04 | -.14 | -.06 | -.06 | -.11 | .13 | .42 | - |  |  |  |  |  |  |  |  |
| 11. Perceived health consequences of zero-COVID | -.07 | .12 | .01 | .05 | -.13 | -.12 | .43 | -.23 | -.37 | .04 | - |  |  |  |  |  |  |  |
| 12. Perceived norms of approval of zero-COVID | -.06 | .08 | -.01 | .03 | -.03 | -.25 | .42 | -.27 | -.43 | -.06 | .57 | - |  |  |  |  |  |  |
| 13. Hope in response to zero-COVID | -.05 | .11 | -.02 | .06 | -.05 | -.18 | .52 | -.29 | -.57 | -.14 | .60 | .69 | - |  |  |  |  |  |
| 14. Support for zero-COVID | -.06 | .08 | -.01 | .07 | -.13 | -.21 | .47 | -.35 | -.53 | -.10 | .61 | .75 | .75 | - |  |  |  |  |
| 15. Perceived health consequences of relaxing restrictions | -.01 | -.01 | -.04 | -.03 | .14 | .28 | -.17 | .11 | .23 | -.05 | -.27 | -.21 | -.19 | -.24 | - |  |  |  |
| 16. Perceived norms of approval of relaxing restrictions | .04 | .03 | -.04 | .02 | .10 | .40 | -.18 | .22 | .28 | -.04 | -.30 | -.37 | -.32 | -.38 | .72 | - |  |  |
| 17. Hope in response to relaxing restrictions | .02 | -.02 | -.03 | -.02 | .17 | .37 | -.20 | .25 | .35 | .00 | -.31 | -.35 | -.34 | -.43 | .68 | .77 | - |  |
| 18. Support for relaxing restrictions | .03 | .02 | -.02 | .00 | .14 | .36 | -.26 | .26 | .37 | .00 | -.34 | -.41 | -.39 | -.45 | .72 | .83 | .83 | - |
| *M* |  | 31.14 | 15.68 | 11.54 | 4.72 |  | 5.86 | 3.73 | 2.99 | 4.41 | 5.96 | 5.87 | 5.71 | 5.71 | 2.68 | 3.03 | 3.34 | 3.06 |
| *SD* |  | 8.40 | 2.44 | 7.82 | 1.41 |  | 0.79 | 1.38 | 1.36 | 1.46 | 0.87 | 1.11 | 1.11 | 1.19 | 1.50 | 1.81 | 1.80 | 1.87 |

*Note. N* = 910. ^*^ *r* > .065 (*p* < .05)

# **Figure S1**. *Path Diagrams of the Variables Predicting Chinese Public Support for Zero-COVID*

Support for zero-COVID

COVID fatigue

Perceived norms of support for zero-COVID

Hope induced by zero-COVID

Fatalistic beliefs about getting COVID-19

-0.16

-0.18

0.11

0.17

0.41

0.34

0.47

-0.29

Health consequences of zero-COVID

0.51

-0.02/-0.11

Support for zero-COVID

Liberty

Perceived norms of support for zero-COVID

Hope induced by zero-COVID

0.41

0.34

Health consequences of zero-COVID

0.19

0.12/-0.01

0.17

0.51

Collectivism

0.47

0.31

0.33

*Note*. *N* = 910. If two numbers are shown on one path, the first is the path coefficient for June 2022 and the second is the coefficient for December 2022. The four antecedent variables were entered in the same models when estimating the path coefficients. For aesthetic purposes, the paths from the four antecedent variables are shown in two panels (and the paths from the mediators to policy support were the same). Path coefficients were unstandardized. Demographic variables and political philosophy were controlled for but not shown above.

# **Figure S2.** *Path Diagrams of the Variables Predicting Public Support for Relaxing Restrictions*

0.10

Support for relaxing restrictions

COVID fatigue

Perceived norms of support for relaxing restrictions

Hope induced by relaxing restrictions

Fatalistic beliefs about getting COVID-19

0.13

0.22

-0.13

0.38

0.41

0.68

Health consequences of relaxing restrictions

0.22

0.75

0.19



Support for relaxing restrictions

Liberty

Perceived norms of support for relaxing restrictions

0.38

0.41

Health consequences of relaxing restrictions

0.14

0.07/-0.11*

-0.13

0.19

0.75

0.13

Hope induced by relaxing restrictions

Collectivism

0.68

-0.17

-0.17/0.08

*Note*. *N* = 910. If two numbers are shown on one path, the first is the path coefficient for June 2022 and the second is the coefficient for December 2022. The four antecedent variables were entered in the same models when estimating the path coefficients. For aesthetic purposes, the paths from the four antecedent variables are shown in two panels (and the paths from the mediators to policy support were the same). Path coefficients were unstandardized. Demographic variables and political philosophy were controlled for but not shown above.
